# Extremely heat tolerant photo-symbiosis in a shallow marine benthic foraminifera

**DOI:** 10.1038/srep30930

**Published:** 2016-08-09

**Authors:** C. Schmidt, D. Titelboim, J. Brandt, B. Herut, S. Abramovich, A. Almogi-Labin, M. Kucera

**Affiliations:** 1 MARUM, Center for Marine Environmental Sciences, University of Bremen, Leobener Str. Bremen 28359, Germany; 2 Department of Geological and Environmental Sciences, Ben Gurion University of the Negev, Beer Sheva, P.O.B 653, Beer Sheva 84105, Israel; 3 National Institute of Oceanography, Israel Oceanographic & Limnological Research, Haifa (IORL), Shikmona, P.O.B. 8030, Haifa 31080, Israel; 4 Geological Survey of Israel, 30 Malkhe Israel St., Jerusalem 95501 Israel

## Abstract

Bleaching, the loss of algal symbionts, occurs in marine photosymbiotic organisms at water temperatures minimally exceeding average summer SST (sea surface temperatures). Pre-adaptation allows organisms to persist under warmer conditions, providing the tolerance can be carried to new habitats. Here we provide evidence for the existence of such adaptation in the benthic foraminifera *Pararotalia calcariformata*. This species occurs at a thermally polluted site in the Mediterranean, where water temperatures reach a maxima daily average of 36 °C during the summer. To test whether this occurrence represents a widespread adaptation, we conducted manipulative experiments exposing this species from an unpolluted site to elevated temperatures (20–42 °C). It was kept in co-culture with the more thermally sensitive foraminifera *Amphistegina lobifera* in two experiments (20–36 °C). Reduced photosynthetic activity in *A. lobifera* occurred at 32 °C whereas photochemical stress in *P. calcariformata* was first observed during exposure to 36 °C. *Pararotalia calcariformata* survived all treatment conditions and grew under 36 °C. The photosymbiosis in *P. calcariformata* is unusually thermally tolerant. These observations imply that marine eukaryote-eukaryote photosymbiosis can respond to elevated temperatures by drawing on a pool of naturally occurring pre-adaptations. It also provides a perspective on the massive occurrence of symbiont-bearing foraminifera in the early Cenozoic hothouse climate.

Photosymbiosis is a widespread strategy in shallow marine ecosystems, particularly among marine calcifiers living in oligotrophic conditions^1^. Under this mutualistic relationship the host consumes the derived ‘photosynthates’ from the symbionts and the symbionts have access to re-mineralized nutrients inside their host, which are higher compared to the surrounding sea water^2^,^3^. Photosymbiosis occurs across a diverse range of hosts, including prolific ecosystem engineers like corals, giant clams and foraminifera. The reef ecosystems that they help to sustain deliver 10^4^ g carbonate m^2^/year^4^ and play essential roles in the maintenance of marine biodiversity^5^. The symbiotic relationship is sensitive to environmental stressors such as elevated temperature, light and salinity^6^,^7^ and nutrients^8^, which can induce symbiont loss of the microalgae or their associated pigments leading to mortality of entire reefs^7^,^9^.

Resistance to elevated temperatures differs among coral species^10^. It is shaped by fine-scale differences in symbiont type^11^ and can be influenced by regional thermal pre-adaptation in areas such as the Red Sea^12^ or the Arabian Gulf^13^,^14^. The bleaching threshold in foraminifera is similar to corals, and ensues when local temperature maxima are exceeded by 1–3 °C 8,15,16,17. Because of recent warming, bleaching thresholds are breached during extreme heat spells in tropical waters at an increasing frequency leading to widespread bleaching events in 1998, 2002 18 and in 2015/2016 19. On the other hand, corals and foraminifera have flourished during greenhouse intervals in Earth’s geological history, such as the early Eocene, when tropical temperatures appear to have been significantly warmer than today^20^,^21^,^22^ and reefs occurred at high latitudes^23^. This would imply that marine organisms living in the most exposed coastal tropical areas must have been able to tolerate temperatures above the current thermal threshold. Notwithstanding this observation, it is unclear whether such adaptations are still present among extant species or whether these species will be able to draw on these adaptations as present climatic change proceeds extremely rapidly^24^.

Here we take advantage of the recent discovery of the occurrence of symbiont-bearing foraminifera at a thermally polluted site (Hadera) on the Mediterranean coast of Israel, where cooling water discharge from a power plant raises local sea surface temperatures by on average 6 °C above the baseline^25^. We use this artificial “window” into a warmer ocean to investigate the thermal tolerance of the benthic foraminifera *Pararotalia calcariformata*. Its thermal tolerances seems to exceed that of Arabian Gulf corals, which possess the highest bleaching threshold worldwide and survive short-term exposure to 36 °C without bleaching^13^,^14^. Further, we investigate if the thermal thershold is especially high at the thermally polluted site (Hadera) by exposing populations from an unpolluted site (Nachsholim) to elevated temperatures in replicated culturing experiments. In parallel, we recorded sea-water temperatures at both sites throughout one year and monitored the foraminiferal population *in-situ*. We measured photosynthetic activity of the symbionts using Pulse Amplitude Modulated (PAM) Fluorometry, and recorded survivorship and growth, as an indicator for holobiont fitness. Demonstrating the representativeness of our laboratory conditions, the first two experiments included populations of *Amphistegina lobifera* from the same locality, a genus which is known to bleach at 32 °C in other locations worldwide^15^,^26^,^27^.

## Results

### Field Observations

**Figure 1:**
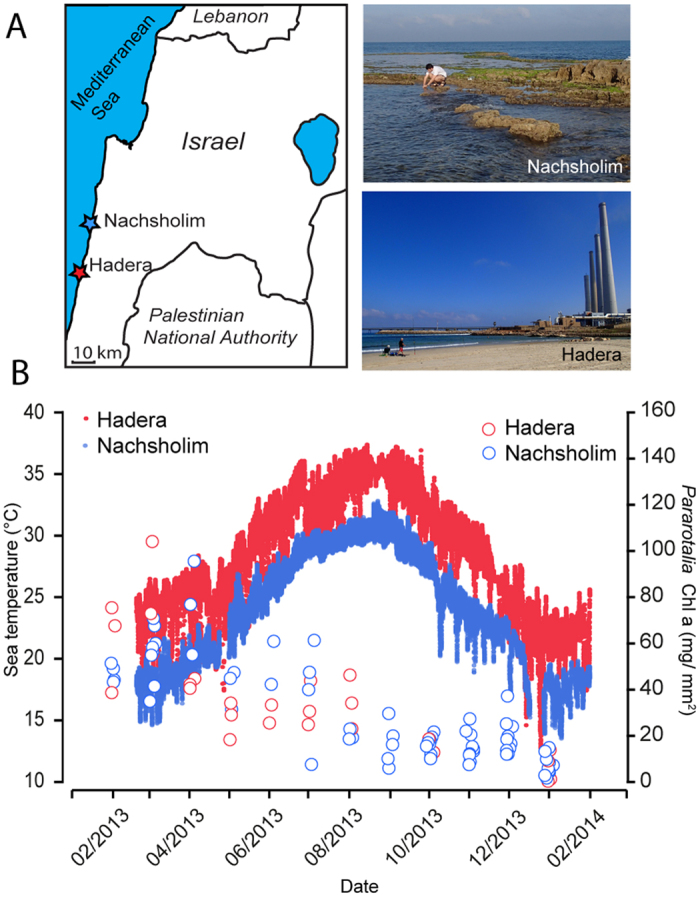
Illustration of the sampling area in the eastern Mediterranean and temperature logger data during the seasonal cycle of 2013. Figure 1A The Levantine Basin and the two sampling sites in this study: the heat-exposed site (Hadera) where cooling water raises locally ambient sea water temperatures, and the control site (Nachsholim) towards the north, Map was created in Adobe Illustrator software re-drawn after OpenStreetMap, Fig. 1B Sea water temperature (dots) measured by loggers at these sites (~0.5 m) during 2013 annual cycle^44^ (left) and chlorophyll a pigment data (mg/mm^2^ surface area) measured in the foraminifera *Pararotalia calcariformata* collected from these locations (circles).

To constrain the temperature range to which the natural populations at the two studied sites are exposed even during short, extreme heat spells, we deployed *in-situ* loggers in Hadera and in Nachsholim recording temperature every 15 minutes between February 2013 and January 2014 (Fig. 1). We found that both locations show similar seasonal cycles, but temperatures above 33 °C never occurred in the natural habitat (Nachsholim) (maximum daily average, 30.8 °C), whilst maximum daily average temperatures of 36.2 °C up to 37 °C were recorded inside the heat-plume (Fig. 1). Field observations also showed that pH levels at the sites stay year-round in the normal marine range of 8.1–8.2 pH. In line with earlier surveys^25^, *P. calcariformata* was found at both stations throughout the year, despite the extreme temperatures at Hadera. To test whether the Hadera population was bleaching, we measured the total chlorophyll a (chl a) content of *P. calcariformata* throughout the monitoring period. The results revealed intact photosynthetic pigment in the species during the seasonal cycle, showing similar values to the population from the unpolluted site (Fig. 1). The values were highest during the spring and early summer and lower but similar at both sites during the rest of the year including late summer thermal maximum and the cold season.

### Experimental Approach

To confirm the extreme heat tolerance of *Pararotalia calcariformata*, populations from the unpolluted site together with the more sensitive *Amphistegina lobifera* (which was absent from the polluted site) were exposed to a temperature gradient from ambient values at time of collection to 36 °C. The experiment was replicated (Fig. 1) for a summer population (SPE) and in a winter population (WPE). The seasonal replication served to account for the effect of pre-exposure (heat hardening during the summer), which is required because of the large seasonal temperature cycle at the studied site (Fig. 1). In addition, a summer population extreme experiment (SPEE) was performed on *P. calcariformata* for temperatures up to 42 °C to determine the bleaching threshold of this species.

### Symbiont Response

**Figure 2:**
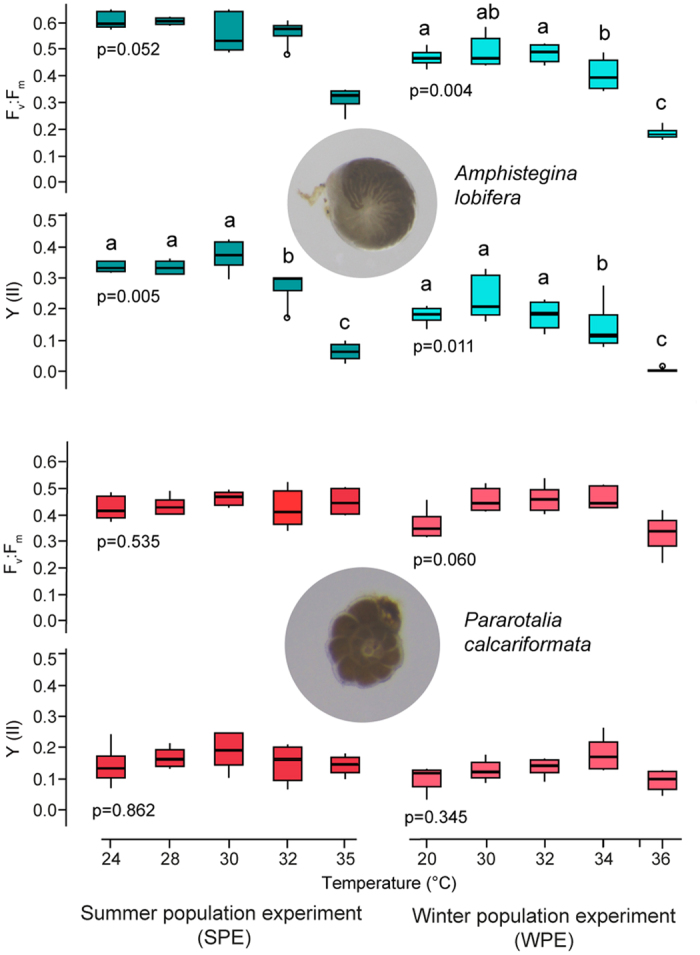
Photochemistry responses in the manipulative experiments of *Amphistegina lobifera* (turquois) and *Pararotalia calcariformata* (pink) after two weeks of exposure to experimental temperatures. F_v_:F_m_ maximum quantum yield, Y(II) effective quantum yield measured at experimental light intensity, data plotted are shown as boxplots (the top and bottom of the box represent 1^st^ and 3^rd^ quartile, the 2^nd^ quartile the median, and the lines extending from the box extent to the outermost data that fall within the distance computed as 3^rd^ quartile ± 1.5 *interquartile range), each graph has N = 29–33 independent data points, which are given as means per well, each well contained N = 5–6 specimens, see Table S1 for statistical analysis.

The photochemical dark yield was measured as maximum quantum yield (F_v_:F_m_) before the start of the SPE (*A. lobifera* 0.618, SD ± 0.031, *P. calcariformata* 0.555, SD ± 0.029), and remained in the controls (24 °C) at the same level over the course of the experiment. PERMANOVA (Fig. 2) revealed that F_v_:F_m_ was not affected by the temperature increase during exposure after two weeks up to 35 °C. The light yield Y(II) decreased in *A. lobifera* after one week exposure, but not in *P. calcariformata* (Table S1). A pair-wise Monte Carlo test showed that 24–30 °C were significantly different from 32 °C and 35 °C (Table S1).

For the WPE the initial photochemical dark yield F_v_:F_m_ was for *A. lobifera* 0.529, SPE ± 0.047 and for *P. calcariformata* 0.451, SPE ± 0.049 and remained the same over the course of the experiment in the controls. PERMANOVA revealed that temperature had a significant effect on F_v_:F_m_ in *A. lobifera* after one week exposure but not in *P. calcariformata,* which showed significant reductions only after three weeks of exposure (Fig. 2, Table S1). The Y(II) was significantly reduced in *A. lobifera* after two,week exposure but not in *P. calcariformata* (Fig. 2) After one week exposure in the WPE photoinhibition (F_v_:F_m_ < 0.01) occurred in *A. lobifera* at 36 °C (Fig. 2).

**Figure 3:**
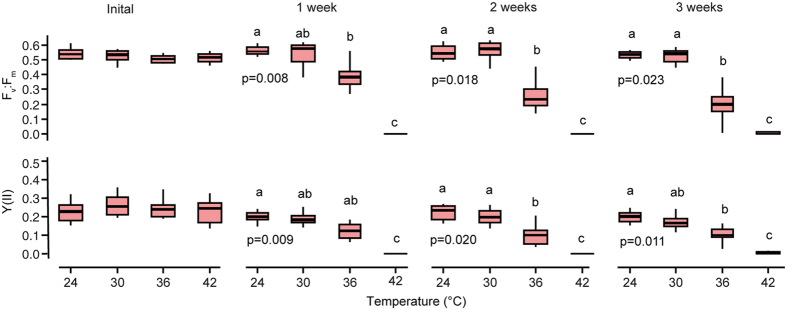
Photochemistry responses in the extreme exposure experiment (SPEE) of *Pararotalia calcariformata* over three weeks to elevated temperatures. F_v_:F_m_ maximum quantum yield, Y(II) effective quantum yield measured at experimental light intensity, data plotted are shown as boxplots (the top and bottom of the box represent 1^st^ and 3^rd^ quartile, the 2^nd^ quartile the median, and the lines extending from the box extent to the outermost data that fall within the distance computed as 3^rd^ quartile ± 1.5 *interquartile range), Each graph has N = 47–48 independent data points, which are given as means per well, each well contained N = 5–6 measurements, see Table S1 for statistical analysis.

Since no signs of bleaching or photoinhibition were observed in *P. calcariformata* at 36 °C, a summer population extreme experiment (SPEE) was conducted exposing this species to 24 °C, 30 °C, 36 °C, and 42 °C to determine its upper thermal tolerance level (Fig. 3). The photochemical dark yield F_v_:F_m_ and light yield Y(II) stayed on the same level in the 24 °C (control) and in 30 °C treatment throughout the experiment (Fig. 3). PERMANOVA revealed that F_v_:F_m_ and Y(II) was significantly negatively affected by temperature after one week of exposure (Fig. 3, Table S1). Monte Carlo post hoc tests indicate that after one weeks exposure there are significant differences between the treatments (Table S1). After one week photoinhibition (F_v_:F_m_ < 0.01) occurred in all wells of the 42 °C treatment and after three weeks in one well from the 36 °C treatment (Fig. 3).

### Holobiont response

**Figure 4:**
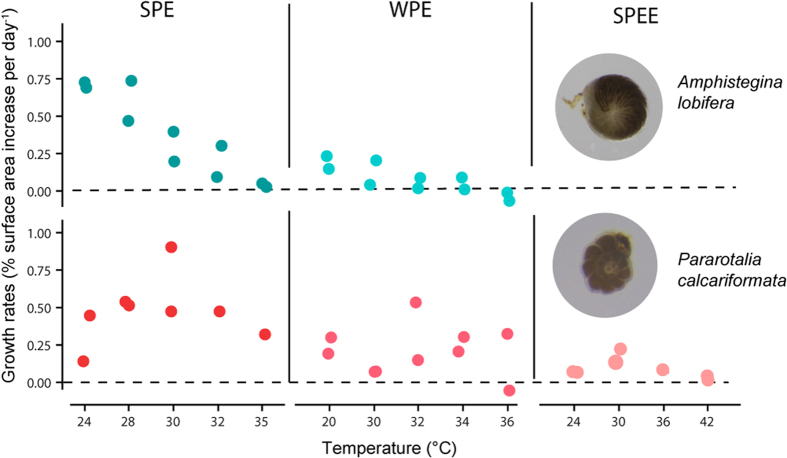
Mean growth rates per aquaria of *Amphistegina lobifera* and *Pararotalia calcariformata* after the manipulative experiments. SPE (summer population experiment), WPE (winter population experiment) and the SPEE (summer population extreme experiment).

Survivorship was high throughout all experiments. Mean survival rates were between 89–100% per treatment (Table S2). Positive mean growth rates (percent surface area increase per day^−1^ per aquaria) were observed in all experiments in the control treatments. *Pararotalia calcariformata* still grew at 35 °C whereas *A. lobifera* showed growth inhibition at this level in the SPE (Fig. 4). The growth rates of *P. calcariformata* under 30–35 °C were within the range of the controls in both seasons (Fig. 4), whereas growth rate of *A. lobifera* was reduced already at 32 °C. In the extreme temperature experiment (SPEE) the summer population of *P. calcariformata* grew less than in the previous experiments. Very low growth rates at 24° and 36 °C and an inhibition of growth in the 42 °C treatment were observed (Fig. 4).

**Figure 5:**
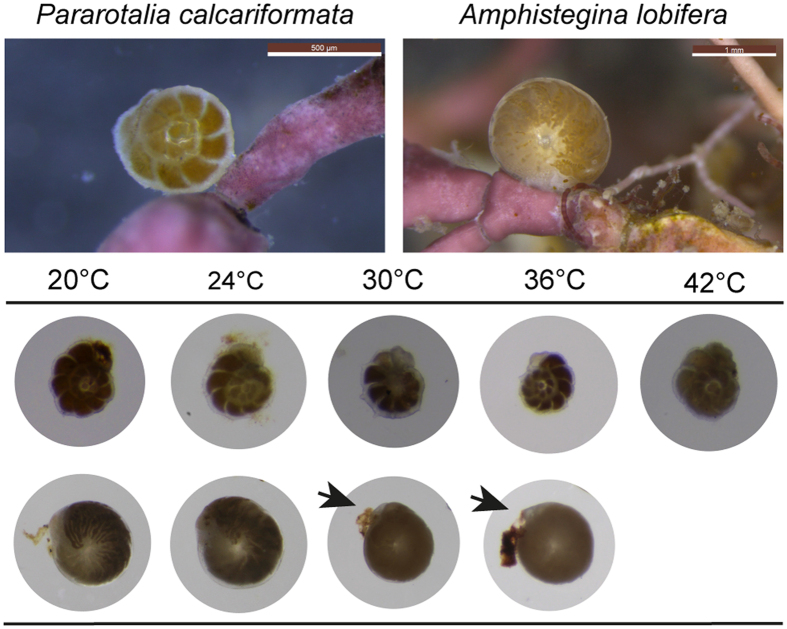
Microscopic images of the species *Amphistegina lobifera* and *Pararotalia calcariformata*. Images show specimen with healthy symbiont coloration in its natural habitat in comparison to healthy and bleached specimens after the exposure to manipulative experiments for two weeks, arrows indicated symbiont loss in *A. lobifera* at temperatures >30 °C.

To illustrate bleaching responses, the cultured specimens were photographed during the exposure experiments (Fig. 5). Cytoplasmic color consistent with symbiont pigment presence was visible in *P. calcariformata* even after three weeks exposure to 42 °C, whereas bleaching occurred in *A. lobifera* at temperatures above 32 °C, as shown by the selected specimen from the 34 °C treatment, where the digested and/or expelled symbionts are visible (Fig. 5).

## Discussion

Our results indicate that the benthic foraminifera *Pararotalia calcariformata* is able to sustain an active photosymbiosis up to 36 °C for at least three weeks. This confirms the field occurrence of this species with intact symbiont pigments at the heat polluted site and indicates that the extreme thermal tolerance is also present in the unpolluted site, where temperatures are normal. This result is supported by the observed sensitivity of *Amphistegina lobifera*, which was exposed to the same temperature gradients and showed evidence of photochemical stress, growth inhibition and bleaching at temperature levels that are in line with previous studies^15^,^27^. The bleaching threshold of *P. calcariformata* seems to be between 36–42 °C, as all specimens at 42 °C showed chronic photoinhibition (F_v_:F_m_ ≤ 0.01), consistent with irreversible damage to Photosystem II 6. Chronic photoinhibition has been observed in corals due to light and temperature stress but at much lower temperatures^28^. In larger benthic foraminifera photoinhibition was measured at temperatures of 33–34 °C^15^,^16^. In *P. calcariformata,* despite chronic photoinhibition after one week, the foraminifera did not visibly digest and/or expel their symbionts after three weeks and appeared to be alive. The results that *P. calcariformata* was observed alive and not bleached in the field despite temperatures of 37 °C and was able to maintain active photosymbiosis after three weeks of constantly elevated temperatures at 36 °C (+/−0.5 °C) in the laboratory show that the thermal tolerance of this species is exceptional. Its thermal tolerance levels appear to surpass the bleaching threshold of Arabian Gulf corals, which are considered the most thermally resistant marine eukaryotic photosymbiotrophs^13^,^14^.

We speculate that the apparently innate tolerance to high temperature of *P. calcariformata* in the studied eastern Mediterranean region originates from the parent populations of this species in the Indo-Pacific, as we have previously identified the species to be a recent invader^29^. In tidal pools of the tropics, *P. calcariformata* may experience temporary exposure to extreme temperatures, which may have induced the evolution of its tolerance. We note that populations of this species exhibiting such tolerance would have had an advantage when passing the shallow coastal areas of the southern Red Sea on their invasion route reaching later the Mediterranean. Such thermal filtering has been proposed to explain elevated resistance of corals from the northern Red Sea^12^. Further, the increased tolerance of invasive species in general may reflect a higher adaptive capacity of invaders exposed to stress at the leading edge of their range expansion^30^,^31^. However, this hypothesis alone does not explain the species-specific differences between *P. calcariformata* and *A. lobifera* in our experiment. Both species are currently expanding in the Mediterranean^29^,^32^ and can thrive in similar coastal habitats (Fig. 5), so they should exhibit similar adaptations and adaptability. Also, it is not clear why short-term exposure to elevated temperature in tidal pools should induce the evolution of tolerance to sustained exposure.

In corals, it has been suggested that thermal tolerance is linked to symbiont type^21^,^33^. The symbiont specificity in foraminifera is different from corals, and next to species hosting *Symbiodinium* like in corals, other species prefer different algal groups, often present in diverse consortia^34^,^35^. *Pararotalia calcariformata* forms a symbiosis with at least three different diatoms, including *Minutocellus polymorphus* (<2–3 μm size), which has not been isolated as symbiont from any other host^29^. *Amphistegina lobifera* has been shown to host up to seven different species of endo-symbiotic diatoms but not *M. polymorphus*^36^. We suggest that the symbiotic association of *P. calcariformata* with *M. polymorphus* could be the clue to the increased thermal resistance of the holobiont. When grown in batch-culture *M. polymorphus* showed threefold increase of anti-oxidant activity (super oxide dismutase activity, SOD) at the beginning of its exponential growth phase^37^. This indicates that *M. polymorphus* may be able to counteract thermal stress in the holobiont through induction of anti-oxidants to the protoplasm.

Benthic foraminifera are prolific carbonate producers and important ecosystem engineers and a major component in the global carbon cycle^38^,^39^. Considering the previously observed thermal thresholds for bleaching in these organisms^15^,^16^,^17^,^26^,^27^, the fate of them in tropical coastal environments under continued warming, is of major concern. Our discovery of a symbiont-bearing foraminifera pre-adapted to extremely high temperatures, as well as its ability to retain this adaptation during invasion indicate that the community composition of tropical foraminifera may change, but the group as a whole and its function are likely to persist in a warmer world. During the late Paleocene to early Eocene global warming larger foraminifera were increasingly favored over coral reefs as the dominant carbonate producing organisms in oligotrophic environments, as their biodiversity, size and abundance increased when Tethyan coral reefs declined^40^,^41^. The potential survival and success of this heat-tolerant species is not certain under global warming, as in recent times both warming and ocean acidification, and other anthropogenic stressors occur in combination, and interactive effects have been shown experimentally on other larger foraminifera^17^,^42^. It seems highly probable that future reefs will see a transition to dominance of certain larger foraminifera, together with algae over corals^43^; however this would lower reef function and biodiversity considerably. Moreover, the existence of naturally occurring high bleaching thresholds in benthic foraminifera has consequences for the understanding of their proliferation^41^ during early Cenozoic hothouse climate. It also means that occurrences of fossil symbiont-bearing foraminifera in tropical settings of the early Cenozoic cannot be used as arguments to contradict reconstructions suggesting hot tropical marine temperatures at that time^20^,^21^,^22^. At least some of these organisms are clearly able to sustain their photo-symbiotic relationship with unicellular algae under temperatures exceeding the present tropical range by 4–5 °C, which could indeed provide this group with a selective advantage in a warmer world.

## Methods

### Field collections

Foraminifera were observed and sampled in a shallow subtidal coastal habitat on the Mediterranean coast of Israel, which represents their typical environment^25^,^29^. The observations were carried out at a heat-polluted site and an unpolluted control site (Fig. 1). Specimens for experiments were sourced from the latter, as *Amphistegina lobifera* does not occur at the heat-polluted station. The heat-polluted station^25^ was located app. 300 m away from the warm water outlet from the Hadera power plant (N32° 27.68167, E 34° 52.95). The unpolluted control station was located app. 18 km north of Hadera, in Nachsholim National Park (32° 37.386 N, 34° 55.169 E), representing natural sea water temperatures in the Levant. At each station, submersible underwater loggers (HOBO, USA) were mounted on bedrock to record ambient temperature in the shallow water habitat (~0.5 m) (19/02/2013 to 31/01/2014, 15 min intervals).

During 2013 and January 2014, each month, field campaigns have been conducted to both stations, sampling living algal material and attached foraminiferal community for monitoring assemblages throughout the year^44^ (2/01, 19/02, 03/04, 14/04, 22/05, 14/06, 30/07, 19/08, 26/09, 17/10, 14/11, 18/12) and in 2014 (7/1). During each sampling campaign, *in situ* water samples were taken to determine temperature, salinity, pH, and dissolved oxygen using a YSI 6600 UPG sampler (please see Supplement 3 of^44^ for further reference). pH levels were recorded at each site during sampling campaigns and stayed in the range of normal occurring marine pH levels of 8.1–8.2.

To determine the total chlorophyll a pigment content of *Pararotalia calcariformata* throughout the year living samples were taken each month. Living specimens were contained in algae mats, which were separated from the bedrock by using a flat knife and placed in small sampling containers (Volume 100 mL) together with seawater. Sampling was conducted at low tide (LAT 0.2–0.5 m). At least 30 specimens were picked shortly after their arrival to the laboratory, cleaned from attached algae and photographed. Samples were divided into groups of ten individuals, which were inserted in Eppendorf tubes and frozen in −80 °C until analysis. Total chl a pigment concentrations were measured on a Kontron (Uvikon) UV 930 Spectrophotometer and normalized to specimen size, following a previous protocol^15^.

### Sample collection for culturing experiments

Living benthic foraminifera for culturing experiments were collected at Nachsholim National Park (32° 37.386 N, 34°55.169 E) attached to filamentous coralline algae by snorkeling (LAT 0.5–1.5 m). Field campaigns for experimental studies sampled two summer populations (1/11/2012, 23/10/2013) and one winter population (08/04/2013) of *A. lobifera* and *P. calcariformata*. Samples were transported in large plastic bottles filled with algae and sediment to the laboratory. There, algae and sediment were immediately rinsed with fresh sea water and specimens were picked from the concentrated sediment. Individually picked specimens (up to 50) have been put inside sea water filled jars together with some sediment material. The jars (volume 150 mL) were put in an insulated container and expressed shipped to the laboratory in Bremen, Germany, where subsequent experiments were conducted. During transport the samples experienced minimal temperature fluctuations, which were recorded by temperature loggers placed in the shipment (HOBO®Onset; USA). They revealed temperature conditions of 22.2–23.2 °C, SD 2.0 °C (duration of shipments 24–36 h). Upon arrival in Bremen, the specimens were cultured inside the jars at sampling water temperature of the collection site under a diurnal light cycle (12 h/12 h, PAR of <30 μmol m^2^ s^2^) in incubation chambers at ambient salinity (38.5–40.0‰). Half of the seawater was replaced every week by at least 30% freshly made seawater (Tropic Marine Sea Salt, Germany) to replace trace metals. Each culture was kept under these conditions for 2–3 weeks before commencing the experiments.

### Culturing experiments

A replicated design consisting of 10 aquaria (volume 18 L) was set up for conducting the summer (SPE) and winter exposure (WPE) experiments exposing both species housed in the same aquaria at five different temperatures for 2–3 weeks. Temperature was manipulated separately using heating rods in each aquaria and externally controlled (AT control, Aquamedic, Germany), which automatically regulated temperatures when it was +/−0.1 °C from the aimed values. As the 20 °C treatment during the winter population experiment (WPE) was below the ambient temperature of the room, two aquaria were externally cooled additionally to the precise heating rods by a water cooler circulating water around the aquaria in a special constructed cooling container using a strong pump (Ocean Runner 1200, Aquamedic) and a water cooler (Titan 150, Aquamedic). Temperatures were set to fall into climate change predictions for the Mediterranean Sea which has seen a general warming over the last decades, which is predicted to continue by up to 0.4–2.8 °C in the near future^45^. Temperatures during the summer population experiment (SPE) were between 24–35 °C and during the winter population experiment (WPE) between 20–36 °C (Fig. 2). We choose to slightly adjust the temperatures and the duration of the experiments focusing during the WPE on the warmer end of the spectrum, taking into account results of SPE. Manual temperature measurements were conducted daily using a handheld temperature and salinity meter (WTW, Germany) to check that set temperatures were kept within the range of +/−0.5 °C from set temperatures. Daylight fluorescent bulbs (50:50 actinic 420 nm/10 K trichromatic, 12 h day/12 h night) provided light for the aquaria at an intensity of 35–40 μmol m^2^ s^2^ (Apogee MQ-200, USA). These values are slightly below the photosynthetic optimum for *A. lobifera*, which have a maximum saturating irradiance at ~80 μmol m^2^ s^2^ PAR based on measurements of relative electron transport rates of their symbionts using PAM Fluorometry when low light adapted^46^. For *P. calcariformata* maximum saturating levels are reached 40 μmol m^2^ s^2^ 29, therefore those have been used for co-culture experiments, as experiments over longer periods are best to be carried out at lower intensities as values derived from light adaptation measurements because specimens can show stress exposed to higher light intensities over longer period of time. For the extreme temperature experiment (SPEE), temperature controlled incubation chambers (Pol-Eko-Aparatura, Model ST2+/ST2+, Poland) were used, as the upper thermal temperature of 42 °C cannot be sustained over weeks in the open aquaria setup. The temperature range for this experiment was set between 24–42 °C (Fig. 3) for the duration of 3 weeks. Manual temperature measurements inside the aquaria were made daily using a handheld thermometer (WTW, Germany) for monitoring: 24 °C treatment = 23.6 °C (SD 0.19), 30 °C treatment = 29.4 °C (SD 0.27), 36 °C treatment = 35.4 °C (SD 0.42), 42 °C treatment = 41.2 °C (SD 0.55). Into each aquaria a small temperature logger (HOBO) was placed recording temperature in 30 min intervals (N = 931). Recordings were as follows: 24 °C treatment, 24.0 °C (SD 0.42), 30 °C treatment 29.9 °C (SD 0.44), 36 °C treatment 36.3 °C (SD 0.53), 42 °C treatment 42.5 (SD 0.65). Each incubation chamber contained two small plastic aquaria (volume 2 L) illuminated with constant light conditions (diurnal light cycle 12 h/12 h, 19–26 μmol m^2^ s^2^, white-fluorescent light bulbs). The treatment water was automatically oxygenated by bubbling air with small hand-held air pumps into the seawater (30 min/day). Sea water was made in bulk using artificial sea salt (Tropic Marin® Sea Salt, Germany) at the beginning of the experiments. Salinity measurements were made daily with the same instrument with which the temperature was checked (WTW, Germany) and was adjusted to stay in the range between 38.5–40.2 ppm in all experiments, by adding deionized water. These levels are generally observed in the Levantine Basin and represent *in-situ* conditions for the experimental species^47^. The pH levels of the seawater were monitored weekly and stayed >8.1 pH units. At the beginning of the experiment and after weekly measurements, foraminifera were fed with marine microalgae *Nannochloropsis* (frozen, autoclaved) by adding 15 μl of standard food mixture to the glass jars, as previously published^29^. Culturing containers consisted of a standard snap-cap top vial (volume 15 mL, Wheaton, UK) with a hole cut in the cap underneath which a small piece of plankton mesh was fixed (*P. calcariformata* mesh size of 100 μm and *A. lobifera* 300 μm). Six snap-cap vials were put inside each aquarium on the bottom of a standard 6 well-plate to ensure stability, containing either 5-6 specimens. After weekly measurements the arrangement of the vials inside the aquaria was randomized to reduce bias due to different positions inside the aquaria, after mesh has been cleaned with water from algae to ensure same light conditions throughout the experiment.

### Response parameters

To carry out photochemistry measurements on PS II, a chlorophyll fluorometer IMAGING-PAM *M-Series* Fluorometer (WALZ GmbH, Germany) was used. It was equipped with “MAXI-Head,1/2” CCD camera and zoom objective (F1.0/f = 8–48 mm). Weekly foraminifera were removed from their treatment vial and transferred into petri dishes with treatment water for PAM measurements. Measurement of dark adaptation (=maximum quantum yield, MQY, F_v_:F_m_) was conducted after 10–20 min followed by light-adapted yield (effective quantum yield, EQY, Y(II)) was measured under light adaptation similar to experimental light levels, supplied by LED lights installed in the MAXI-Head (25 μmol photos m^2^ s^2^) recorded by a PAR lightmeter (Apogee, USA). To measure 4–6 specimens at once, we elevated the petri dishes closer to the zoom objective on a 1.5 cm-high stand on the Leaf Holder IMAG-MIN/BK (WALZ, Germany) for *P. calcariformata*. Description on further data processing is given in previous work on foraminifera using the IPAM^15^,^16^,^17^. To record survivorship and growth rates, high resolution photographs were taken at the start, after two weeks and after three weeks of the experiment (Canon SLR camera, Zeiss V8 stereomicroscope). Survivorship was defined whether or not foraminiferal cytoplasma had visible brownish color observable on high resolution photographs taken by stereomicroscopy. This non-terminal method was chosen, as the species have distinct cytoplasm color which can be distinguished from dead individuals, which becomes translucent upon cell death^48^. Growth rates were calculated as growth rates per aquaria after the formula from previous studies^15^. Aquaria and wells (WPE) were excluded from the data set where more specimens than initially inserted where recovered, due to the movement of a few specimens, to eliminate bias. We observed asexual offspring in few aquaria during the experiment, resulting in death of the mother individual and negative growth rates, which have been removed from the data set. Missing and dead specimens were removed from the data sets by sorting after diameter size and by checking individual features (unevenness) of specimens.

### Statistics

Statistical analyses of photochemistry (Figs 2 and 3) are based on means per experimental unit (well) to avoid pseudoreplication. Photochemistry means (F_v_:F_m_, Y(II)) were log (x + 1) transformed because they represent proportions. PERMANOVA–permutational ANOVA (Primer v6, Add-on) was performed on transformed data, as it can be used as a better ANOVA. ANOVA assumes normal distribution, whereas PERMANOVA uses permutations to make the data distribution free and works with any distance measure. Temperature has been included as a fixed factor and aquaria (temp) were included as a random factor to account for variances between the aquaria. Monte Carlo post hoc tests were performed when PERMANOVA was overall significant (p < 0.05) for the factor temperature to establish differences between the individual temperature treatments (Table S1). Growth data per aquaria (% surface area increase per day) was not statistically evaluated as it presents a small sample size (means per aquaria, n = 1–2), and did not meet the criteria of normality assumptions.

## Additional Information

**How to cite this article**: Schmidt, C. *et al.* Extremely heat tolerant photo-symbiosis in a shallow marine benthic foraminifera. *Sci. Rep.* **6**, 30930; doi: 10.1038/srep30930 (2016).

## Supplementary Material

Supplementary Information
