# Supplementary material for: Extremely heat tolerant photo-symbiosis in a shallow marine benthic foraminifera
Source: Sci Rep. 2016 Aug 9;6:30930. doi: 10.1038/srep30930 (PMC4977509; doi:10.1038/srep30930)
Supplement: Supplementary Information [file srep30930-s1.pdf]

Supplementary information

Extremely heat tolerant photo-symbiosis in a shallow marine benthic foraminifera

Schmidt, C.[a,1], Titelboim, D[b], Brandt, J.[a], Herut, B.[c], Abramovich, S.[b], Almogi-Labin, A.[d], and Kucera, M.[a,1]

[a]MARUM, Center for Marine Environmental Sciences, University of Bremen, Leobener Str. Bremen 28359, Germany

[b]Department of Geological and Environmental Sciences, Ben Gurion University of the Negev, Beer Sheva, P.O.B 653, Beer Sheva 84105, Israel

[c]National Institute of Oceanography, Israel Oceanographic & Limnological Research, Haifa (IORL), Shikmona, P.O.B. 8030, Haifa 31080, Israel

[d]Geological Survey of Israel, 30 Malkhe Israel St., Jerusalem 95501 Israel

Tab. S1 PERMANOVA on photochemistry variables (F_v:F_m and Y(II)) in the manipulative temperature experiments, data was log (x+1) transformed prior to analysis species names and factor names are abbreviated: P=*Pararotalia calcariformata*, A=*Amphistegina lobifera,* T=Temperature, Aq=Aquarium, pair-wise Monte Carlo post-hoc tests were performed when $p<0.05$ on the factor temperature, sig. p values are underline

Experiment	Week	Species	Factor	PERMANOVA Maximum Quantum Yield (Fv:Fm)				
			Source	df	MS	F-ratio	p	pair-wise Monte Carlo test
1. Summer Population Experiment 24, 28, 30, 32, 35°C (SPE)	1	*A*	T	4	81.537	49.888	0.048	A, AB, AB, AB, C
			Aq (T)	5	1.6344	0.44319	0.819	
			Error	20	3.6879			
		P	T	4	7.9329	0.56614	0.716	
			Aq (T)	5	14.012	2.3153	0.079	
			Error	20	6.0522			
	2	*A*	T	4	772.51	31.141	0.052	
			Aq (T)	5	24.825	1.6816	0.177	
			Error	19	14.763			
		P	T	4	19.965	0.93899	0.535	
			Aq (T)	5	21.262	1.0633	0.405	
			Error	20	19.997			
2. Winter population experiment 20, 30, 32, 34, 36°C (WPE)	1	*A*	T	4	5127.1	13.959	0.005	A, AB, B, AC, D
			Aq (T)	6	367.3	0.78154	0.688	
			Error	22	469.97			
		P	T	4	262.03	2.6932	0.089	
			Aq (T)	6	97.295	7.4323	0.001	
			Error	22	13.091			
	2	*A*	T	4	1853.9	110.09	0.004	A, AB, A, B, C,
			Aq (T)	6	16.84	0.91052	0.487	
			Error	22	18.495			
		P	T	4	299.7	4.492	0.06	
			Aq (T)	6	66.718	2.8863	0.026	
			Error	22	23.115			
	3	*A*	T	4	2269.5	71.716	0.002	A, A, AB, B, C
			Aq (T)	6	31.645	1.3145	0.28	
			Error	22	24.074			
		P	T	4	145.68	6.9668	0.019	AB, AB, A, AB, B
			Aq (T)	6	21.069	1.6593	0.18	
			Error	20	12.698			
3. Extreme Temperature Experiment 24, 29, 35, 41°C (SPEE)	1	*P*	T	3	15586	11.957	0.008	A, AB, B, C
			Aq (T)	4	1303.5	1.1047	0.295	
			Error	39	1180			
	2	*P*	T	3	17899	13.593	0.018	A, A, B, C
			Aq (T)	4	1316.8	1.0047	0.42	
			Error	40	1310.6			
	3	*P*	T	3	17360	13.724	0.023	A, A, B, C
			Aq (T)	4	1264.9	0.87742	0.599	
			Error	40	1441.7			

Experiment	Week	Species	Factor	PERMANOVA Effective Quantum Yield (Y(II))				
			Source	df	MS	F-ratio	p	pair-wise Monte Carlo test
1. Summer Population 24, 28, 30, 32, 35°C (SPE)	1	*A*	T	4	331.76	44.232	<u>0.021</u>	A, B, B, AB, C
			Aq (T)	5	7.5005	1.1361	0.366	
			Error	20	6.6018			
		P	T	4	274.85	1.6913	0.313	
			Aq (T)	5	162.51	1.414	0.251	
			Error	20	114.93			
	2	*A*	T	4	4755.1	114.22	<u>0.005</u>	A, A, A, B, C
			Aq (T)	5	41.46	0.29827	0.959	
			Error	19	139			
		P	T	4	210.21	0.35051	0.862	
			Aq (T)	5	599.71	5.0004	<u>0.005</u>	
			Error	20	119.93			
2. Winter population 20, 30, 32, 34, 36°C (WPE)	1	*A*	T	4	6775.3	6.442	<u>0.005</u>	AB, A, A, B, C
			Aq (T)	6	1051.7	1.1719	0.253	
			Error	22	897.44			
		P	T	4	1066.8	3.7915	0.058	
			Aq (T)	6	281.37	1.511	0.222	
			Error	22	186.21			
	2	*A*	T	4	6667.1	6.6206	<u>0.011</u>	A, A, A, B, C
			Aq (T)	6	1007	0.97988	0.495	
			Error	22	1027.7			
		P	T	4	836.25	1.2802	0.345	
			Aq (T)	6	653.22	3.4157	<u>0.009</u>	
			Error	22	191.24			
	3	*A*	T	4	6840.5	8.2283	<u>0.008</u>	A, A, AB, B, C
			Aq (T)	6	831.34	0.98589	0.488	
			Error	22	843.24			
		P	T	4	853.63	3.6103	0.057	
			Aq (T)	6	238.94	2.2342	0.079	
			Error	20	106.95			
3. Extreme Temperature 24, 29, 35, 41°C (SPEE)	1	*P*	T	3	15823	9.7948	<u>0.009</u>	A, AB, AB, C
			Aq (T)	4	1615.7	1.3263	0.146	
			Error	39	1218.2			
	2	*P*	T	3	18005	13.306	<u>0.02</u>	A, A, B, C
			Aq (T)	4	1353.2	0.95593	0.489	
			Error	40	1415.5			
	3	*P*	T	3	1.53E+04	15.625	<u>0.011</u>	A, AB, B, C
			Aq (T)	4	979.6	0.86036	0.6	
			Error	40	1138.6			

Tab. S2 Mean survivorship (%) per treatment during temperature sensitivity experiments exposing *Pararotalia calcariformata* and *Amphistegina lobifera* for 2-3 weeks to elevated temperatures, n.e. not exposed

Experiment	Temperature (°C)	Species	
		Pararotalia calcariformata	*Amphistegina lobifera*
Summer Population (SPE) 24, 28, 30, 32, 35°C	24	100	100
	28	100	100
	30	100	100
	32	95	93
	35	93	90
Winter Population (WPE) 20, 30, 32, 34, 36°C	20	100	100
	30	96	90
	32	90	96
	34	89	98
	36	100	98
Extreme Exposure Experiment (SPEE) 24, 29, 35, 41°C	24	100	n.e.
	30	99	n.e.
	36	99	n.e.
	42	99	n.e.
